# Near-infrared spectroscopy muscle oximetry of patients with postural orthostatic tachycardia syndrome

**DOI:** 10.1142/S1793545818500268

**Published:** 2018-08-13

**Authors:** Parvathi Kadamati, Jeffrey J. Sugar, Brendan J. Quirk, Shima Mehrvar, Gisela G. Chelimsky, Harry T. Whelan, Thomas C. Chelimsky, Mahsa Ranji

**Affiliations:** * Biophotonics Laboratory, University of Wisconsin Milwaukee, Department of Electrical Engineering and Computer Science, 3200 N Cramer St., Milwaukee, WI 53211, USA; † Medical College of Wisconsin, Department of Neurology, 8701 W Watertown Plank Rd, Milwaukee, WI 53226, USA; ‡ Medical College of Wisconsin, Department of Pediatries (Gastroenterology), 8701 W Waterown Plank Rd, Milwaukee, WI 53226, USA

**Keywords:** Hemoglobin, head-up tilt table, oxygenation, blood volume

## Abstract

Postural orthostatic tachycardia syndrome (POTS) is a disabling condition characterized by orthostatic intolerance with tachycardia in the absence of drop-in blood pressure. A custom-built near-infrared spectroscopy device (NIRS) is applied to monitor the muscle oxygenation, noninvasively in patients undergoing incremental head-up tilt table (HUT). Subjects (6 POTS patients and 6 healthy controls) underwent 30 mins of 70°on a HUT. The results showed a significant difference in deoxyhemoglobin (Hb), change-in-oxygenation (ΔOxy) and blood volume (ΔBV) between patients and healthy controls. However, oxyhemoglobin (HbO_2_) showed a significantly faster rate of change in the healthy controls during the first 10 mins of the tilt and during the recovery. This NIRS muscle oximetry tool provides quantitative measurements of blood oxygenation monitoring in diseases such as POTS.

## 1. Introduction

Postural orthostatic tachycardia syndrome (POTS) is a type of chronic orthostatic intolerance, annually affecting around 500,000 young Americans.^1,2^ Symptoms of POTS include lightheadedness and persistent increase in heart rate (>30 bpm in adults and >40 bpm in children) with upright body posture.^1–3^ Patients with POTS may experience many other symptoms such as fatigue, sweating, tremors, anxiety, heart palpitation and exercise intolerance.^1–5^ Assuming a recumbent or supine position typically improves symptoms, which may lead to a high level of functional disability.^4,5^ A wide range of medications and treatment strategies are available, mainly emphasizing cardiac volume expansion and exercise.^5^

In this study, the lower muscle oxygenation of POTS patients was monitored to assess the effect of the postural change during a head-up tilt table (HUT). HUT is a standard test for patients with a potential POTS diagnosis to evaluate their response to postural change. During the HUT, blood pressure and heart rate are measured continuously.^6^ However, subjects with an increase in heart rate that meets the diagnostic criteria for POTS may have the same symptoms as those who do not, often termed \orthostatic intolerance”.^7,8^ Thus, other physiological abnormalities may in fact be critical to the development of orthostatic symptoms, which could be detectable with a device such as near-infrared spectroscopy (NIRS), which evaluates deeper aspects of muscle and brain physiology. As a first step, we aimed simply to evaluate whether subjects with POTS showed clear differences from healthy control subjects. An affirmative answer to this question would encourage further studies that might separate patients with POTS from those with orthostatic intolerance without POTS.

NIRS is a noninvasive optical technique that utilizes wavelengths in the near-infrared window (700–1300 nm) to determine the *in vivo* concentrations of chromophores. NIRS oximetry is a viable method for inexpensively monitoring oxygen consumption in muscles and has been widely used to examine hemodynamics of skeletal muscle.^9–11^ Previous studies using NIRS oximetry have reported impaired cerebral oxygenation of patients with POTS.^6^ However, the pathophysiology remains unclear and research is required to understand the underlying conditions that lead to POTS.

The custom-made device can monitor the change in concentrations of the chromophores oxyhemoglobin (HbO_2_) and deoxyhemoglobin (Hb) based on the measured optical densities at certain wavelengths 735 nm and 850 nm.^12,13^ These wavelengths are the absorbance peaks of Hb and HbO_2_ in the near-infrared window.^14^ A third wavelength near the isosbestic point of Hb and HbO_2_ (805 nm), where the absorption of the two chromophores is equal, was used to find the change in combined concentrations of Hb and HbO.^15^

In this study, the NIRS oximeter monitored the lower muscle oxygenation of 6 POTS patients as well as 6 control subjects in our neuroscience research center during HUT test, and the trends were compared to the results from a commercially available NIRS monitor which can only provide oxygenation change (Covidien INVOS 5100C). Muscle oxygenation can provide important information regarding blood circulation and muscle functionality.^16^ Determining the muscle oxygenation of POTS after a physical intervention could potentially aid in the development of diagnostics and treatments that would mitigate the adverse effects of POTS and facilitate the recovery of tachycardia. In this study, the key relationship between impaired muscle oxygenation and POTS is investigated.

## 2. Materials and Methods

### 2.1. NIRS Oximetry device

**Fig. 1.**
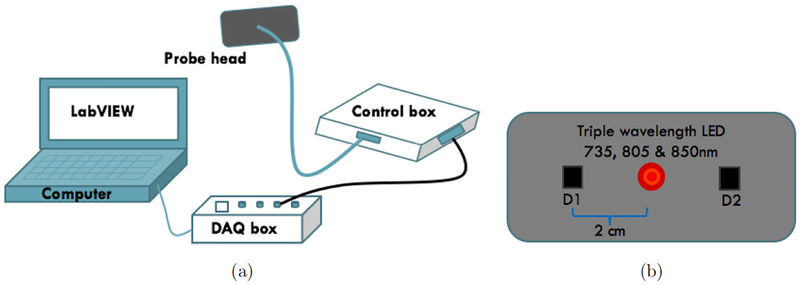
(a) Custom-built NIRS Oximetry system consists of a control box and a probe connected to a laptop through a DAQ box. (b) The probe head geometry consists of a triple wavelength LED and two detectors, arranged in a line with a 2 cm source-detector separation.

The custom-built NIRS oximetry system [Fig. 1(a)] includes a probe head connected to a control box that controls the electro-optic components of the probe and records the real-time data. The control box consists of LED drivers and instrumentation for the detectors. The control box communicates with a digital acquisition board (DAQ board from National Instruments) simultaneously in both analog and digital channels, which is connected to a computer with LabVIEW software (National Instruments) interface.

The probe can produce and detect the reflected signal of three separate wavelengths of light from the tissue, simultaneously. The probe head as shown in Fig. 1(b) consists of an LED light source and photodetectors module. The light source is one triple wavelength LED (L735/805/850/PD-35B32, Epitex, Kyoto, Japan). This LED has a plastic Fresnel lens that collimates the beam. On either side of the source, two multi-pixel photon counters (S1133–14, Hamamatsu, Kyoto, Japan) are placed as detectors. These components are embedded in a silicone head to ensure that the distance between the source and detectors D1, D2 is fixed at 2 cm.

### 2.2. Monte Carlo simulation

The optimal distance between the source and detector depends on the structure of the tissue, the wavelengths used and the desired depth of penetration. Increasing the source to detector separation results in a greater depth of penetration.^10^ However, increasing the separation distance decreases the signal-to-noise ratio. A modified Monte Carlo method is used to choose the optimal source-detector separation for the geometry of calf muscle oximetry.

**Fig. 2.**
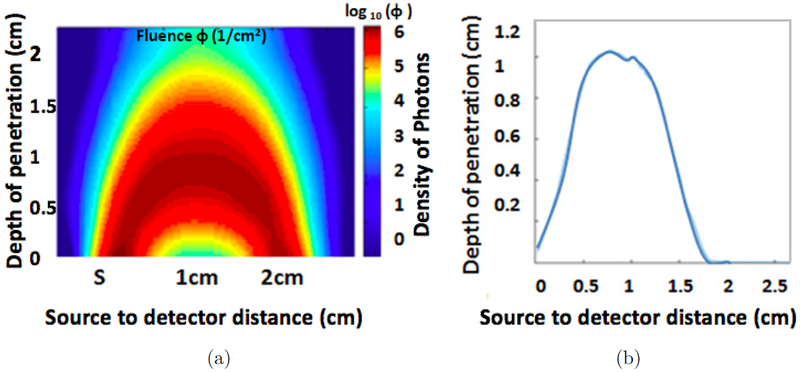
(a) The photon propagation in Monte Carlo simulation for 2 cm source to detector separation. (b) The average effective photon path for the simulation.

The 3D Monte Carlo simulation is used to simulate photon distribution (∅) in a multi-layer heterogeneous model of the tissue. The tissue is modeled by 0.3 mm of the dermis, 1 mm of subdermis and a semi-infinite layer of the muscle beyond. Figure 2(a) illustrates Monte Carlo simulation results of light penetration in the tissue for 2 cm source to detector separation obtained by simulating the trajectories of 10^8^ photons through the tissue. Figure 2(b) shows the densest parts of these simulations to give a sense of the average optical path and the average depth of penetration. The 2 cm source-detector separation gives a depth of penetration up to 1 cm.

### 2.3. Data analysis

In NIRS oximetry, near-infrared light is used to measure the changes in the concentration of Hb and HbO_2_ via the modified Beer–Lambert law as

(1) $$\Delta \text{A}=-\text{log}\left(\frac{I\left(t\right)}{I_{o}}\right)=\Sigma L\mu_{\lambda_{i}}\Delta C_{i},$$

where ΔA refers to a change in attenuation, *I*_*o*_ is the intensity of incident light and *I(t)* is the intensity of detected light measured at some time *t*. *L* is the average optical path length that the photons take from the source to the detector, Δ*C*_*i*_ is the change in concentration of the ith given chemical and *μ*_*λi*_ is the absorption coefficient of the given chemical at a certain wavelength equal to λ.

The three wavelengths can be used to calculate the amount of HbO_2_ and Hb by solving the following systems of linear regression equations:

(2) $$\left\{\begin{array}{c} \Delta \text{A}\left(735\;\text{nm}\right)=L\left(\mu_{\text{Hb}}\left(735\;\text{nm}\right)\Delta C_{\text{Hb}}+\mu_{{\text{HbO}}_{2}}\left(735\;\text{nm}\right)\Delta C_{{\text{HbO}}_{2}}\right)\\ \Delta \text{A}\left(850\;\text{nm}\right)=L\left(\mu_{\text{Hb}}\left(850\;\text{nm}\right)\Delta C_{\text{Hb}}+\mu_{{\text{HbO}}_{2}}\left(850\;\text{nm}\right)\Delta C_{{\text{HbO}}_{2}}\right)\\ \Delta \text{A}\left(805\;\text{nm}\right)=L\left(\mu_{\text{Hb}}\left(805\;\text{nm}\right)\Delta C_{\text{Hb}}+\mu_{{\text{HbO}}_{2}}\left(805\;\text{nm}\right)\Delta C_{{\text{HbO}}_{2}}\right) \end{array}\right\},$$

where Δ*C*_Hb_ and $\Delta C_{{\text{HbO}}_{2}}$ are the change in concentration over time. *μ*_HbO_(λ) and $\mu_{{\text{HbO}}_{2}}\left(\lambda \right)$ are molar extinction coefficients of Hb and HbO_2_ at wavelength equal to λ.^12^ This system can be solved for three wavelengths to find the change in concentrations of Hb (Δ*C*_Hb_) and HbO_2_ $\left(\Delta C_{{\text{HbO}}_{2}}\right)$.

The obtained changes in the concentrations of Δ*C*_Hb_ and $\Delta C_{{\text{HbO}}_{2}}$ are used to calculate the change-in-oxygenation (ΔOxy) and blood volume (ΔBV)^16–18^ as follows:

(3) $$\Delta \text{Oxy}=\Delta C_{{\text{HbO}}_{2}}-\Delta C_{\text{Hb}},$$

(4) $$\Delta \text{BV}=\Delta C_{{\text{HbO}}_{2}}+\Delta C_{\text{Hb}}.$$

Throughout the results section, negative ΔOxy implies that the muscle uses oxygen at a faster rate than can be supplied. Oximetry signals are acquired from the capillary bed that feeds the muscle. A decrease in ΔOxy therefore reflects changes in both HbO_2_ and Hb.^10,15^

The averages of HbO_2_, Hb, ΔOxy and ΔBV were used both to make a quantitative oxygenation comparison between POTS patients versus healthy controls and for statistical analyses. The data were compared using the student two-tailed t-test with a significant difference criterion *p* < 0.01. Linear correlation between our costume-built device and Covidien probe was evaluated in MS Excel.

### 2.4. Subjects

All the POTS patients and healthy controls were recruited under the approval of Institutional Review Board in the University of Wisconsin–Milwaukee and Medical College of Wisconsin (IRB# 16.246, 558266). Six POTS patients (age 8–18 years) and six healthy control subjects (age > 18) underwent the HUT protocol. Individual biometrics of these subjects is detailed in Tables 1 and 2.

The POTS patients showed a rise in heart rate (HR) >40 bpm in first 10 mins of tilt as displayed in Table 3. The healthy control subjects were questioned before the procedure whether they were on any medications, or had no history of fainting, or lightheadedness with standing in the last year.

### 2.5. Experimental protocol

The NIRS oximetry probe was placed on the right gastrocnemius and a standard Covidien oximetry probe (INVOS 5100B, Covidien/Medtronic, Minneapolis MN, USA) was placed on the left gastrocnemius to be used as a reference for comparison of ΔOxy measurements.

**Fig. 3.**
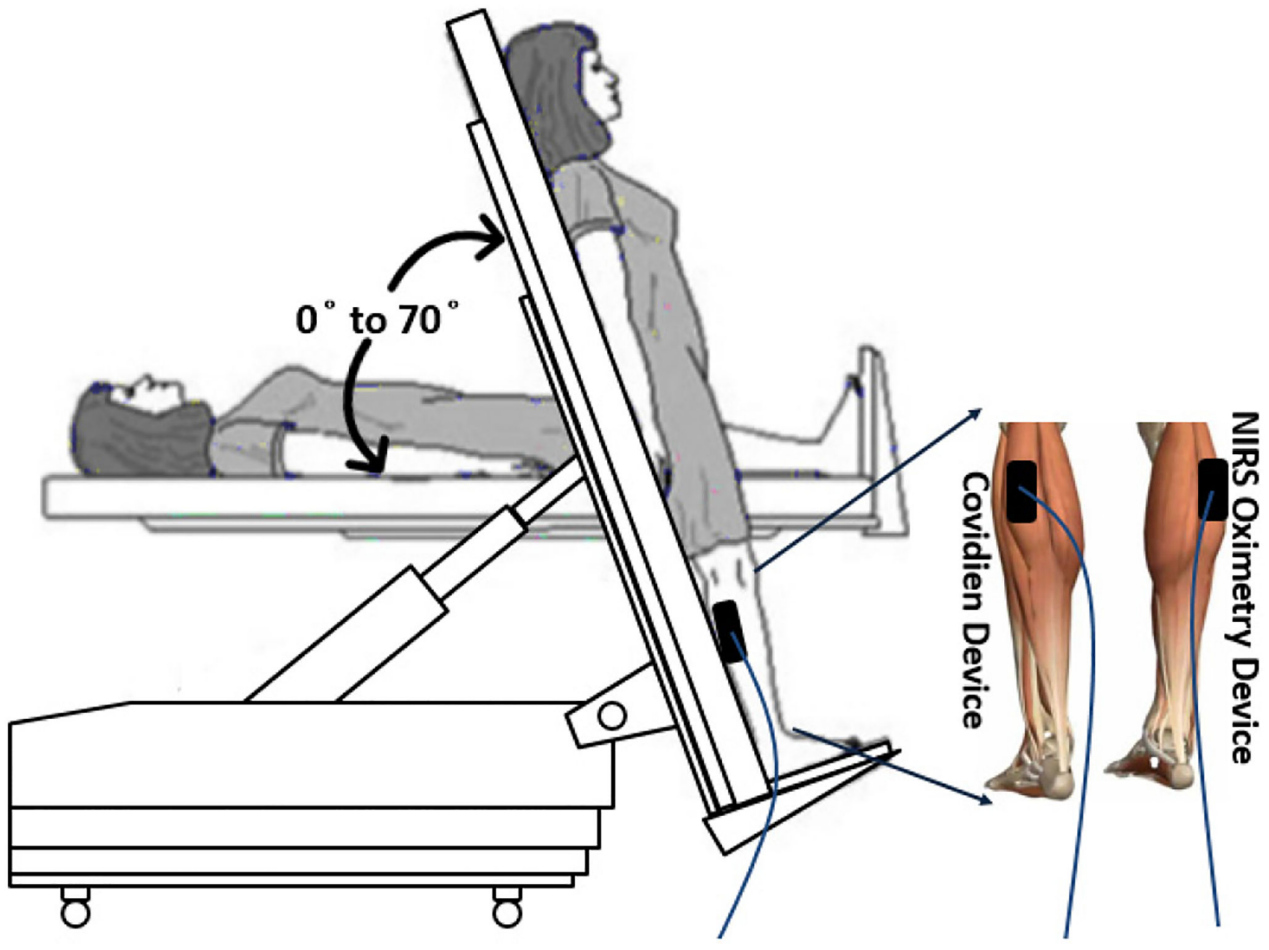
The head-up tilt table (HUT) protocol; NIRS oximetry probe and Covidien probe are attached to the gastrocnemius muscle. The subjects were monitored at 0°, 70° and back to 0° angles of the tilt table for 10 mins at baseline, for 30 mins at a 70° tilt and for 10 mins at recovery.

The subject was asked to lay on the tilt table, monitored for 10 mins at 0° angle of the HUT as the baseline. Monitoring continued for 30 mins after they were taken to 70° over 5 s, and reclined to 0° for 10 mins to assess their recovery as shown in Fig. 3.

## 3. Results

### 3.1. Validation of device

**Fig. 4.**
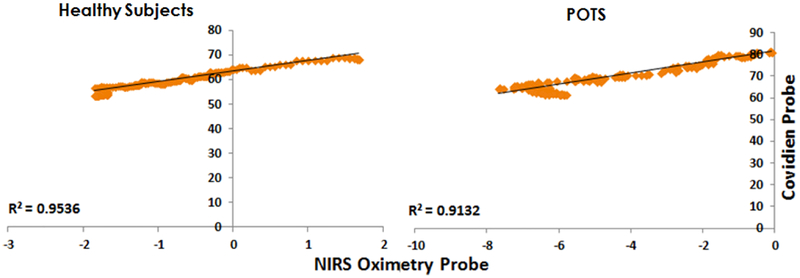
Regional ΔOxy correlation graph between Covidien probe and NIRS oximetry probe results in POTS and healthy subjects during the tilt at 70°. *R*^2^ on the graphs closer to 1.0 show the better fit of the regression line.

As a reference for our custom-built NIRS oximetry probe, the Covidien probe was used to cross-check the ΔOxy. Figure 4 shows the data comparison of the ΔOxy obtained from the custom-built NIRS probe device and the Covidien oximetry device for healthy and POTS subjects. Regional ΔOxy parameter for Covidien oximetry device is \percentage” (%), whereas for the NIRS probe device ΔOxy parameter is \change in concentration” (microMolar or *μ*M).

During the 70° tilt, the data trend is similar to the ΔOxy of both Covidien oximetry device and NIRS probe device for healthy subjects and POTS patients. The value of *R*^2^ (0.95 and 0.91) shows the high correlation of the regression line between Covidien probe and custom-built NIRS oximetry probe.

### 3.2. Physiological findings

**Fig. 5.**
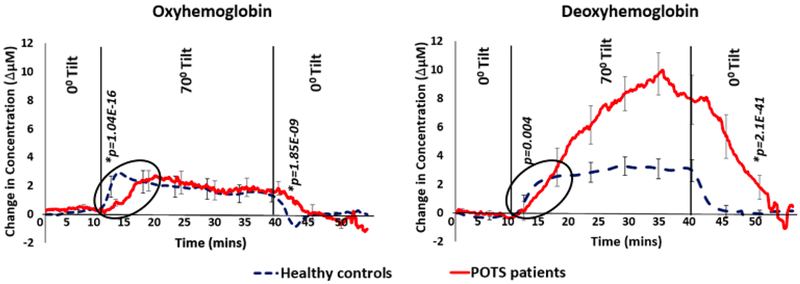
Data comparison between healthy subjects and POTS patients depicts changes in average HbO_2_ and Hb concentrations on changing the position of the tilt table at 0°, 70° and 0° for 10 mins, 30 mins and 10 mins, respectively. POTS subjects displayed a significantly higher increase in Hb, while the change in HbO_2_ was statistically similar in both groups. The *p*-value (*p* < 0:01 significant difference) was determined between the rising and dropping slopes of both the groups for HbO_2_ and Hb.

Figure 5 shows average HbO_2_ and Hb data collected from the healthy control subjects and patients with POTS. The three segments show the change in the posture of the subject from 0° (baseline) to 70° (tilt) and back to 0° (recovery).

**Fig. 6.**
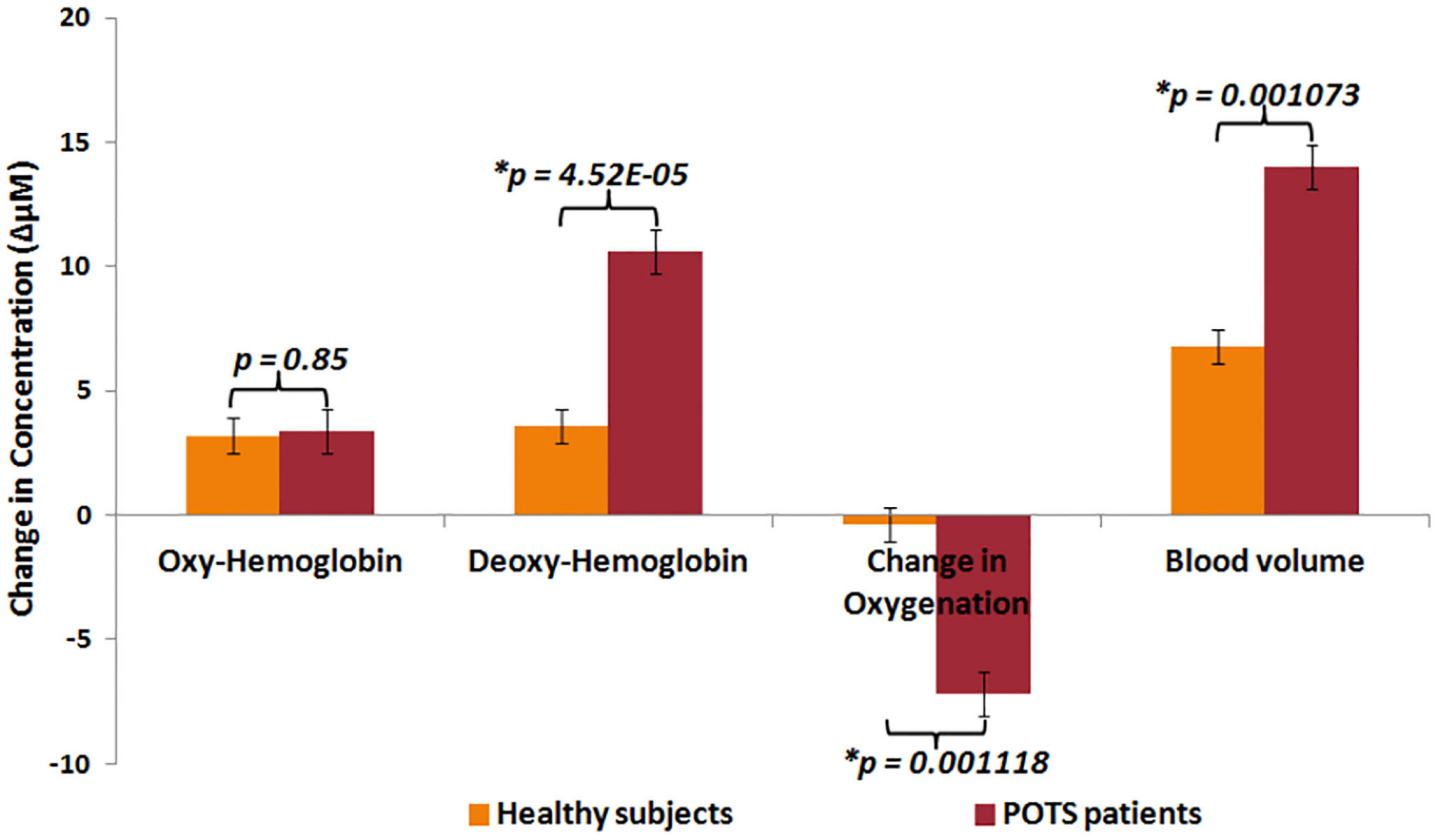
Statistical analysis of healthy subjects and POTS patients. The two groups were compared in average HbO_2_, average Hb, ΔOxy and ΔBV during the 70° tilt. Referring *p*-value < 0:01 shows that there was a significant difference between healthy and POTS subjects (*n* = 6 per group).

The HbO_2_ slightly increased during the 70° tilt in healthy subjects and Hb increased during the same time. For POTS patients, the direction was similar, i.e., increase in Hb and a slight increase in HbO_2_, but the rise in Hb concentration in POTS patients was significantly greater than the rise in healthy subjects (see Fig. 6 for statistics).

The rates of change in HbO_2_ and Hb were analyzed during the 10 mins after the tilt was initiated and the subject was returned to a supine position. A significant difference in the rate of change of HbO_2_ was observed between the POTS patients and healthy control group. The rates of change were tabulated and tested for significant difference using a two-tail Student t-test. This test resulted in significant difference (*p* < 0.01) rate of change in HbO_2_ immediately after the tilt was initiated and after it was stopped. Hb only showed significant difference after the tilt was ended. In Fig. 5, the *p*-values for comparing the rates were also presented.

The area under the curve (units: Δ*μ* M.mins) was calculated during the 30 mins of tilt. The average area under the HbO_2_ curve in POTS was 42 and in healthy controls was 41. The average area under the Hb curve in POTS was 113 and in healthy controls was 52. The HbO_2_ curve area did not vary significantly, while the area under Hb for POTS was double the area under the healthy controls Hb signal.

### 3.3. Statistical analysis

In Fig. 6, each bar represents the averages of the HbO_2_, Hb, ΔOxy and ΔBV for both groups of POTS and healthy subjects. The values represent the mean ± SE during the 70° tilt.

HbO_2_ between patients and healthy controls did not show a statistically significant difference. Hb showed a significant difference between patients and healthy subjects. The observed difference showed that the Hb signal was significantly higher in patients with POTS than in healthy controls, while the two groups had a similar HbO_2_ level in their muscle.

ΔOxy between patient and healthy controls showed a significant difference. The drop of ΔOxy concentration in patients was significantly greater than in healthy control subjects. Also, ΔOxy changes in POTS patients was significantly more than healthy controls.

## 4. Discussions and Conclusions

This study investigated the potential use of NIRS for the quantitative measurement of calf muscle oxygenation in POTS patients. Using noninvasive NIRS of the calf muscle, HbO_2_ and Hb were measured and the ΔOxy and ΔBV were calculated. The reflectance NIRS system used a differential form of the modified Beer–Lambert law to relate the change in concentration of each of the chromophores to the change in optical densities at each wavelength. The custom-built probe was designed to be used on muscle geometry applied to study the oxygenation change in the calf muscle of patients suffering from POTS during a change in posture with head-up tilt table. To the best of our knowledge, this study is the first to investigate the impact of POTS on muscle oxygenation impairment that ensues during the postural change.

This study could potentially help in determining whether tissue tonicity plays a role in the symptoms experienced by POTS patients. Such results could be used to quantitatively track the progress of a therapy in terms of changes in muscle oxygenation. Another application would be in POTS examinations, as a means of providing objective evidence of the degree of the syndrome, which adds a new dimension to what can be measured. It would be interesting to look at subjects who have orthostatic intolerance without POTS and determine if they also have the same abnormality.

The muscle oximetry results revealed that POTS patients and controls showed similar hemodynamics during 10 min baseline measurements (Fig. 5) explaining that without a postural change, there was no hemodynamic impairment between POTS affected patients and control subject. A similar trend observed at the end of the recovering period showed that even though POTS patients might have some hemodynamic impairment during the postural change, after assuming a supine position, their hemodynamics could eventually recover to their baseline. However, during the recovery period, the recovery of HbO_2_ and Hb were slower in POTS patients compared to controls suggesting either a delay in muscle oxygenation recovery of POTS patients or such magnitude of departure from normal that recovery takes significantly longer.

After the postural change to 70° tilt, all subjects experienced an increase in both oxy and Hb because the postural change caused venous blood to pool in the lower extremities. The rate of change was about the same in all subjects, but the healthy controls did not increase after 4 mins in contrast to 30 mins in the POTS group (Fig. 6). The area under the Hb curve in POTS was twice than the healthy subjects curve area. That implies the increase in ΔBV in POTS patients. The higher Hb in POTS patients during 70° tilt could be because of both impaired blood flow and increased oxygen consumption. The impaired blood flow is consistent with some of the tachycardia symptoms such as a drop-in blood pressure in upper torso and head or cerebral hypo-perfusion. However, during the 70° tilt, there was no significant difference in hemoglobin levels between the two groups suggesting that the rate of oxygen consumption in POTS patients were similar to healthy controls. These findings were compatible with venous pooling and poor veno-constriction, as generally hypothesized for POTS pathophysiology.^3^

The remarkable differences in the pattern of change in HbO_2_ and Hb may provide a new understanding of the underlying pathophysiology of POTS (Fig. 5). Healthy subjects showed a sharp rise in HbO_2_ that was quickly halted within the first 2 mins upright, while in POTS patients took about 10 mins to rise to the same level of HbO_2_. Thus, the rate of change in HbO_2_ differed markedly between the two groups. This finding suggests that the POTS subjects did not appear to stop accumulating Hb during the tilt, which is highly suggestive of venous pooling. After the tilt ended and the subjects reclined, a similar phenomenon was observed. During the 10 mins after the tilt, Hb trend in healthy controls decreased at a faster rate than it did in POTS subjects. The slower rate in POTS subjects could be explained by either having less oxygenated blood or having trouble clearing the blood from their extremities that collected due to venous pooling.

These differences suggest that there was some active initial critical process rapidly responding to the upright position in healthy subjects, with the reverse occurring after reclining, that failed in POTS patients. Possible explanations might include: (1) rapid accumulation of arterial blood in a tissue compartment such as arteriovenous (AV) shunts in muscle or skin beds on tilt-up (perhaps serving as a vasoconstrictive signal) and the reverse on recline in healthy subjects, which failed to occur in POTS patients; (2) noncompliant arteries of POTS subjects because they were already maximally constricted or dilated and therefore had minimal ability to store blood in this compartment in response to the upright posture; (3) absence of the presumed central nervous system reflex that produced this response (i.e., some issue with the baroreflex proper); (4) change in the fascial structure in or around the muscle beds, that negatively affected arterial compliance and prevented rapid expansion or constriction of vessels; and (5) oxygenation of the tissues in POTS was inadequate (compared to healthy subjects) and the increase in leg blood with standing was immediately extracted so that HbO_2_ did not rise.

The regional ΔOxy as measured by Covidien probe INVOS 5100 C also showed a precipitous decline during the tilt that mirrors the ΔOxy values from the NIRS oximeter. The average ΔOxy signal between the NIRS oximetry and Covidien device shows the similar trend in all the subjects presenting a significant correlation between the two devices. The study showed (1) expected increase in venous blood was presumably based on the increase in Hb,(2) more interestingly, unexpected rapid upstroke of HbO_2_ was seen in healthy subjects and was absent in the POTS patients, suggesting a new explanation for POTS pathophysiology. The slopes of normal compliance confirm Stewart’s group findings about normal venous compliance.^19^ In POTS patients, we found that the arterial system was less compliant with the inability to receive additional blood volume, explaining the slower response rates observed.

In conclusion, the custom-built NIRS showed a capacity to quantitatively analyze changes in Hb and HbO_2_ leading to new insights about POTS. A device with this ability would have many applications in the clinical settings for monitoring blood oxygenation in patients. Future work should aim to determine whether the calf muscle activation of POTS patients is comparable to that in the healthy controls. The hypotheses could be further explored by imaging the muscle in the upright compared to supine positions. Such results would help to determine whether tissue tonicity plays a role in the symptoms experienced by POTS patients.

## Figures and Tables

**Table 1.** POTS patient’s data.

POTS patients	Age	Gender	Height (cm)	Weight (kg)
1	14	F	175.7	58
2	13	F	157.5	41.1
3	16	F	168	63
4	15	F	159.9	75.3
5	11	M	149.9	44
6	16	F	167	51.6

**Table 2.** Healthy subject’s data.

Healthy subjects	Age	Gender	Height (cm)	Weight (kg)
1	22	M	170.6	64
2	21	M	173	65
3	18	F	145.3	51
4	22	F	158.9	60
5	21	F	155.4	55
6	23	M	182.8	63

**Table 3.** Subject’s heart rate changes.

Subject type	Max average HR during baseline	Max HR during tilt (bpm)	Tilt duration (mins)
POTS	76	123	30
Healthy	66	85	30
